# Co‐Designing a Palliative Dementia Care Framework to Support Holistic Assessment and Decision Making: The EMBED‐Care Framework

**DOI:** 10.1111/hex.70011

**Published:** 2024-08-31

**Authors:** Nathan Davies, Elizabeth L. Sampson, Jesutofunmi Aworinde, Juliet Gillam, Charlotte Kenten, Kirsten Moore, Bethan Phillips, Catherine Harvey, Janet Anderson, Jane Ward, Catherine J. Evans, Clare Ellis‐Smith, EMBED‐Care Team

**Affiliations:** ^1^ Centre for Ageing Population Studies, Department of Primary Care and Population Health, Royal Free Campus University College London London UK; ^2^ Centre for Psychiatry and Mental Health, Wolfson Institute for Population Health Queen Mary University of London London UK; ^3^ Marie Curie Palliative Care Research Department University College London London UK; ^4^ Department of Psychological Medicine, Royal London Hospital East London NHS Foundation Trust London UK; ^5^ Department of Palliative Care, Policy and Rehabilitation, Cicely Saunders Institute King's College London London UK; ^6^ Department of Medicine, Royal Melbourne Hospital The University of Melbourne Melbourne Victoria Australia; ^7^ National Ageing Research Institute Parkville Melbourne Australia; ^8^ Faculty of Medicine, Nursing and Health Sciences, Central Clinical School Monash University Melbourne Victoria Australia

**Keywords:** co‐design, decision making, dementia, palliative care

## Abstract

**Background:**

People with dementia have complex palliative care needs that are often unmet, including physical and psycho‐social needs. It is essential to empower people with dementia, family carers and professionals to better assess and manage care needs. We aimed to co‐design a palliative dementia care Framework delivered through a digital app to support holistic assessment and decision making for care in the community and care homes—the EMBED‐Care Framework.

**Methods:**

A systematic co‐design approach was adopted to develop the EMBED‐Care Framework across three stages: 1) Framework analysis to synthesise data from preceding evidence reviews, large routine clinical data and cohort studies of unmet palliative dementia care need; 2) Co‐design using iterative workshops with people with dementia, family carers and health and social care professionals to construct the components, design of the app and implementation requirements; and 3) User testing to refine the final Framework and app, and strengthen use for clinical practice and methods of evaluation.

**Results:**

The Framework was co‐designed for delivery through an app delivered by aTouchAway. It comprised five main components: 1) holistic assessment of palliative care needs using the Integrated Palliative care Outcome Scale‐Dementia (IPOS‐Dem); 2) alert system of IPOS‐Dem scores to highlight unmet needs; 3) IPOS‐Dem scores and alerts enable shared decision making between the practitioner, patient and/or carer to support priority setting and goals of care; 4) evidence‐informed clinical decision support tools automatically linked with identified needs to manage care; and 5) Training package for users incorporating face‐to‐face sessions, clinical champions who received additional face‐to‐face sessions, animated videos and manual covering the main intervention components and email and telephone support from the research team.

**Conclusions:**

This is a novel digital palliative dementia care intervention to link holistic assessment with clinical decision support tools that are practical and easy to use but address the complexity of palliative dementia care. The Framework is ready for feasibility testing and pilot studies for people with dementia residing at home or in a care home.

**Patient or Public Contribution:**

We were guided by our Patient and Public Involvement (PPI) group consisting of three people with mild dementia, including younger onset dementia, and seven family carers throughout the project. They supported the overall development of the Framework, including planning of workshops, interpreting findings and testing the framework in our PPI meetings.

**Abbreviations**
DEEP: Dementia Engagement and Empowerment Project
Embed‐Care: Empowering Better End‐of‐Life Dementia Care
IPOS‐Dem: Integrated Palliative care Outcome Scale‐Dementia
NHS: National Health Service
PPI: Patient and Public Involvement

## 1 Background

Worldwide, there are 55 million people living with dementia and this number will grow exponentially to 78 million in 2030 and to 139 million in 2050, with population ageing [1, 2]. Dementia is the most common cause of death in the United Kingdom [3] and a global public health priority. Dementia is a progressive terminal condition that benefits from a palliative care approach [4]. However, many people with dementia have complex palliative care needs that they cannot communicate with dementia progression and are often unrecognised and unmet. Palliative care is a holistic approach to care that aims to increase the quality of life for those living with life‐limiting conditions through addressing physical, psychological, social and spiritual needs [5]. A palliative approach can be of benefit throughout the dementia trajectory and should take a needs‐based approach, not limited by time or prognosis [6]. Despite only a third of people living until the advanced stages of dementia, most interventions to support palliative dementia care have focussed on the advanced stages of dementia or those at the end of life [7, 8].

Many people with dementia have multiple long‐term conditions and take several medications, which all require a personalised holistic approach to care and support [9, 10]. Core to personalised care and person‐centred care is understanding the individual needs of the person; this includes ensuring that people can stay in their usual place of care and have timely access to skilled care in the community to meet increasing needs with progression of dementia and/or comorbidities. This is particularly important for people with dementia residing at home or in a care home, and who are at risk of decline with disease progression and unplanned hospitalisation with nearness of end of life [11, 12].

Personalised care should also ensure that people with dementia are included in decisions about their own care, through a process of shared decision‐making. However, people with advancing dementia are too often excluded from assessment processes and discussions about priorities and goals of care [13]. Assessment of needs should be holistic and person‐centred to understand what matters to the person (and the family) and maximise quality of life and support for the person and those around them. Family carers and professionals often struggle to assess, monitor and manage individual care. The Integrated Palliative Care Outcome Scale for People with Dementia (IPOS‐Dem) is a person‐centred outcome measure [14] developed to support holistic assessment of symptoms and concerns, covering physical, psychological, spiritual and social needs. The IPOS‐Dem was adapted the IPOS, which has been validated and widely used in clinical practice [14].

Identification of unmet needs from holistic assessment can leave staff and family cares feeling uncertain about how to prioritise, manage or support needs [6]. Many people with dementia will not have an advance care plan in place and it is not possible to plan for the wide range of symptoms and scenarios that may occur towards end of life; contexts and situations continually change [15]. Professionals and family carers have expressed the need for more support when caring for people with dementia, in particular with managing uncertainty and making decisions about care [16, 17, 18]. Decision frameworks have been developed to support decision‐making among family carers and professionals, including decisions aids [19, 20, 21] and heuristics/rules‐of‐thumb clinical decision support frameworks [22, 23]. However, these are not supported by holistic assessment and so they may aid decisions in the moment but lack a full consideration of an individual's concerns, which they may not be able to express. There is a necessity for linkage of comprehensive needs‐based assessment with comprehensive decision support.

One approach is linkage of these different components of assessment and decision‐making through digital interventions to support the delivery, for example, via an application or website. The COVID‐19 pandemic has accelerated the use of technology to deliver health and across services globally [24, 25]. Digital interventions have been successfully used in other long‐term conditions, including diabetes [26], and in palliative care symptom management for people with advanced cancer [27].

This study is part of a larger programme of work Empowering Better End‐of‐Life Dementia Care (EMBED‐Care), which aims to deliver a step change in care through a large sequential study, spanning multiple work streams [28]. Studies within EMBED‐Care include evidence synthesis and policy reviews, prospective cohort studies, big clinical data analysis, co‐design of a Framework to promote integrated palliative dementia care and feasibility/pilot studies. The aim of this paper is to describe the co‐design process to construct the EMBED‐Care Framework delivered through a digital app, for holistic needs assessment, with linked clinical decision support tools and training package for use by people with dementia, family carers and health and social care professionals in the community and in care homes. This is important to report clearly, as there is a dearth of literature that provides in depth the process of conducting co‐design to develop complex interventions and other co‐approaches with older adults [29].

## 2 Methods

### 2.1 Design

A systematic and iterative partnership co‐design approach working with people with dementia, family carers and health and social care professionals was adopted to develop the EMBED‐Care Framework [30]. Co‐design approaches have been used previously for intervention development in dementia care [20, 21, 23, 31]; this ensures that the voices of those with lived experiences and those working with people with dementia are heard and included in the design and development [30].

### 2.2 Underpinning Theory

We followed the Medical Research Council (MRC) guidance for developing complex interventions [32] and drew on several theoretical frameworks and conceptual models to guide the development of the EMBED‐Care Framework. We developed a logic model based on findings from earlier EMBED‐Care studies [28], including reviews of the literature [33, 34, 35], cohort study and big clinical data analysis [11, 36], of what good integrated palliative dementia care looks like. The logic model was used as the theoretical lens to inform the EMBED‐Care Framework and refined following the co‐design workshops [37]. We drew on existing theory and models to inform the Framework, including person‐centred care, comfort, shared decision making and integrated palliative care [38, 39].

### 2.3 Underpinning Evidence

In earlier studies of EMBED‐Care, we conducted a series of systematic reviews to gather the evidence base to underpin the Framework [33, 34, 35]. We mapped service‐level models of palliative dementia care to identify key components that included person‐centred care, holistic assessment, promotion of well‐being, optimal symptom management and education for family carers and staff. A review of individual‐level interventions across the domains of psychosocial, physical and spiritual care provided information on effective interventions and treatments to inform clinical decision support tools. A review dedicated to the implementation of eHealth in long‐term care provided key recommendations for our Framework, with lessons applicable for community settings [34]. Finally, a review of person‐centred outcome measures to facilitate shared decision‐making shed light on how our assessment component could be used by multiple stakeholders, including people with dementia, and how to shape shared decision‐making processes [34]. Alongside these reviews, we conducted a review of national and international policy [40]. We drew on findings from earlier published cohort studies on unmet palliative dementia care needs [11, 36, 41]. A prospective cohort study of 85 people with advanced dementia highlighted the day‐to‐day presence of chronic and persistent symptoms, indicating key unmet palliative dementia care needs [41]. Symptoms and needs covered a range of physical and psychological issues, including agitation, apathy, pain, dyspnoea, swallowing difficulties and pneumonia. Most care was provided by general practitioners, with little specialist healthcare, and increased use of social care in the last month of life. The EMBED‐Care retrospective cohort study found that rates of hospital admissions increased as people approached the end of life, suggesting the need for improved community care for people with dementia approaching the end of life [11].

### 2.4 Synthesis of Evidence

We iteratively synthesised evidence using a matrix and approaches adapted from framework analysis. From key national and international policy guidelines, we devised a list of 12 core palliative dementia care domains (see Box 1) [4, 42, 43, 44]. These domains formed a matrix that was used to map and synthesise the evidence from preceding the EMBED‐Care studies and earlier cohort studies (see Table 1 for example matrix). This allowed us to highlight unmet palliative care needs and gaps for each of these domains in the literature to be explored in co‐design workshops. We iteratively revised the 12 core domains encompassing all evidence, including items from the IPOS‐Dem. A separate matrix was constructed with the aim of operationalising how the 12 domains could be met, map to the IPOS‐Dem and include findings from the co‐design workshops. We created this second matrix to allow transparency and create new columns that focussed on components of the Framework, including elements of the clinical decision support tools (see Table 2 for example matrix).

Box 1 **12 core palliative dementia care domains.**

**Table:** 

1. Diagnosis and prognosis 2. Person‐centred care 3. Assessment, goal setting, decision making, advance care planning and review 4. Continuity of care and care co‐ordination 5. Information and education of patients and family 6. Medical management: Assessment and management of comorbidities (e.g., falls, pain, diabetes, incontinence and sensory impairment) 7. Managing behavioural and psychological symptoms of dementia 8. Promoting well‐being and functions (includes spirituality, independence, cognition and activities of daily living) 9. Staff training 10. Comfortable and supportive environment 11. Managing care transitions 12. Support for carers (beyond information needs)

**Table 1:** Example matrix of Domains mapped to evidence of unmet needs, interventions and policy recommendations.

Twelve core palliative dementia care domains/Framework requirements	Evidence of unmet need (Cohort and big data) [41]	Possible interventions for addressing the need (identified from reviews, cohort studies and co‐design)	Are interventions recommended in NICE dementia guidance (1) or EAPC white paper (2)	Operationalise in Framework as
Medical management: Assessment and management of comorbidities (e.g., falls, pain, diabetes, incontinence, sensory impairment)	Cohort data demonstrate high symptom burden, including very high risk of pressure sores (53% of the cohort), pain at rest (11%) and movement (61%), high prevalence of movement disturbances (50%), difficulty swallowing (42%), weight loss (34%) and breathing difficulties (47%). Urinary tract infection is the most common acute medical event, with septicaemia and pneumonia in 17% of those who died.	Holistic assessment—through person‐centred outcome measures (e.g., IPOS‐Dem) [14]. Approaches to maximise comfort over maximal survival with a goal of comfort [45]. Advance directives to minimise unwanted interventions [46]. Environmental assessment and modification with prescription of assistive devices where necessary, problem‐solving strategies to optimise performance in activities of daily living (ADLs), carer education and carer skills training to support the carer to provide optimal care [47]. Focus on understanding the preferences of the individual, their history, needs and abilities. This may include training staff on empathy [48].	1.7 Managing noncognitive symptoms. Conduct structured assessment before treatment to check for environmental or clinical causes, e.g., pain. 1.8.3 Consider using a structured observational pain assessment tool alongside a self‐reported tool and a standardised assessment tool. Repeat pain assessment if the patient appears to be in pain. 1.13 Staff training and education. Provide training to staff around understanding, reacting to and helping people with dementia experiencing pain. 2) Domain 1. Applicability of palliative care. Palliative care to be applied to all treatment, care, symptoms, comorbidities and health problems (1.4) Domain 6. Avoiding overly aggressive, burdensome or futile treatment. Medication for chronic conditions to be reviewed regularly. Antibiotics appropriate with the goal of increasing comfort, though consider life‐prolonging effects (6.2 and 6.6). Domain 7. Optimal treatment of symptoms and providing comfort. Holistic approach to all symptoms. Integrate caregivers to help distinguish between sources of discomfort. Use of tools to assess pain/discomfort/behaviours encouraged. Pursue both pharmacological and nonpharmacological treatment for all symptoms as needed. (7.1, 7.2, 7.3 and 7.4)	Holistic assessment through the IPOS‐Dem and clinical decision support tools of various physical problems

**Table 2:** Example matrix of Framework components.

	Input			Output	
Framework content	Co‐design recommendations	Evidence from earlier matrix	Additional information/resources	Key components in clinical decision support tool	IPOS‐Dem items information supports
Overarching principles of palliative dementia care	There are many factors and considerations that should be woven throughout all decisions, and this could be reflected in an overarching principles tool. For example, quality of life (QOL) is important but not a decision; instead, QOL should be a factor to consider across all decisions. Creating a tool covering overarching principles such as QOL is important to have at the start of the clinical decision support tools that supports not just decision‐making but also assessment. Key topics should include: Getting to know the person. Reassurance of different approaches (i.e., trial and error). Communication challenges. Use of communication aids. Physical and social environment.	Focus of many interventions was on the use of verbal skills (e.g., language choice, speech rate, tone, volume) and/or nonverbal skills (e.g., eye contact, posture, gesture, smile) to perform various speech functions in regular interactions with people with dementia. Targeting support for communication between the individual and others, and supporting positive social interaction are important. An example of this was reminiscence and life story work.	Alzheimer's Society—This is me leaflet. British Geriatrics Society (BGS)—End of Life Care in Frailty: Advance Care Planning. BGS—End of Life Care in Frailty: Community settings. BGS—End of Life Care in Frailty: Care homes. Dementia UK—Tips for better communication	Communication vital throughout and should consider: Talk (including getting to know the person whether through them or their family). Have they understood? Consider the individual's culture, including their language spoken. Consider comfort with assessment of the environment: Setting Lighting Temperature Other people	Have all practical problems been addressed? [e.g., hearing aids, foot care, glasses, diet] Difficulty communicating Has s/he been able to interact positively with others (e.g., staff, family, residents)?

### 2.5 Participants

Co‐design workshops consisted of people with dementia, family carers, health and care professionals and academic experts. Family carers were purposively sampled monitoring age, gender and ethnicity/language/culture/religion to maximise the diversity of the sample and enable a better understanding of the subtlety of issues and decisions, as well as how different people with dementia may approach various decisions. We purposively sampled professionals with a range of roles that would make up a multidisciplinary team caring for people with dementia and their families, including general practitioners, admiral nurses, old age psychiatrists, community nurses, occupational therapists, speech and language therapists, home care workers, nursing home workers, nursing home managers and palliative care doctors and nurses. We invited people with an interest in digital health, managers from National Health Service (NHS) England, managers and commissioners from primary and acute care services and private companies. Professionals were recruited from across settings and disciplines, including social care (home care workers, care home workers), specialists (dementia care, palliative care and geriatrics) and generalists (community and primary care). For specific inclusion and exclusion criteria, see Table 3.

**Table 3:** inclusion and exclusion criteria.

Inclusion	Exclusion
People with dementia	
Clinical diagnosis of any type of dementia (as confirmed by the participants themselves or their family carer)	Unable to provide informed written consent
Capacity to provide informed consent	
Ability to read and speak English	
Family carers	
Current or former carer of someone with dementia	Had a cognitive impairment themselves
Capacity to provide informed consent	Unable to communicate in English
Able to read and speak English	
Professionals	
A caring role, either health or social care, for people with dementia or expertise in dementia and palliative care	
Capacity to provide informed consent	
Were able to read and speak English	

### 2.6 Recruitment

To recruit people with dementia, we worked closely with the Dementia Engagement and Empowerment Project (DEEP), who host a series of regular workshops and meetings for people with dementia, and joined these groups when available. These groups were kept small to ensure that people with dementia felt confident and able to contribute. Our previous work has demonstrated that smaller groups work better with people who have dementia [20].

We recruited family carers using a variety of sources:

1. Approaching participants who took part in earlier phases of the overarching project who indicated in their consent form that they were interested in engaging in future aspects of the study.
2. Carer networks and organisations known to us, palliative care, ageing and dementia care groups and networks, charity and third sector groups and networks. Potential participants were identified by the organisations' preferred method of contact.
3. Join Dementia Research (JDR): an online self‐registration service that enables volunteers to register their interest in taking part in research.
4. Newsletters, magazines and websites of leading dementia and ageing organisations and outlets.
5. Social media, including X (formerly Twitter) and Facebook, posting short messages and, where possible, posting participant information sheets and or an advertisement poster.

We identified professionals using a variety of sources:

1. Professionals via known contacts of the research team and snowballing methods.
2. NHS sites who took part in earlier phases of the overarching project*.* NHS sites who were taking part in earlier phases were asked to identify professionals and they were invited by their line manager at the NHS organisation. Staff or their manager then contacted the research team directly.
3. Care home staff and carers identified via the Maudsley Biomedical Research Centre (BRC) Care Home Research Network and National Institute for Health and care Research (NIHR) Enabling Research in Care Homes (ENRICH) Networks. The research team contacted potential care homes directly and the manager of the care homes invited potential staff. Interested staff or their manager contacted the research team directly.
4. Home care staff were identified via local and national home care organisations using the Care Quality Commission (CQC) website. We approached the organisations directly and managers within the organisation identified and invited staff. Staff or their manager then contacted the research team directly.

### 2.7 Workshops

We conducted eight workshops with people with dementia, family carers and health and social care professionals. The aims and an overview of each of the workshops, participants and content are summarised in Table 4. These workshops were supplemented with four additional workshops that focussed on co‐designing an implementation plan [49], exploring the use of person‐centred reported outcome measures to support shared decision‐making and specific groups with home care (*n* = 2) and care home (*n* = 2) workers. These two groups are seldom heard and we wanted to maximise their participation in a separate group where they may feel more confident. People with dementia were included in two workshops that were smaller in size to ensure that their voice was heard and to minimise cognitive overload, as recommended by previous work [20]. As the people with dementia included were part of an existing group that met regularly themselves, we had to fit our workshops around their schedule, which meant that we were not able to meet as much as we would have liked. We were also keen to meet people with dementia in person and this was limited due to COVID‐19 regulations. We took an iterative approach to development, with workshops building on the previous one and developing our vision and the prototype Framework, with participants providing constructive feedback on our intervention from early ideas through to a finalised version.

**Table 4:** Overview of co‐design workshops.

Workshop	Aim	Format	Contents
Workshop 1: Professionals (*n* = 31) and carers (*n* = 10)	Define palliative dementia care needs and the focus of the EMBED‐Care Framework.	Online	Introduction of concepts of palliative care and palliative dementia care, IPOS‐Dem assessment and clinical decision support tools. Discussion of palliative care needs guided by case studies/scenarios. Discussion of key features and content for the EMBED‐Care Framework.
Workshop 2: Professionals (*n* = 23) and carers (*n* = 14)	Refining the focus of the clinical decision support tools. Identify the core information for each tool and identify evidence to be used. Explore how the holistic assessment and clinical decision support tools could be linked and used by the person with dementia, family carers and professionals.	Online	Content of the clinical decision support tools, considering the overall structure of the tools and the content of individual topics based on practical experience. How can the IPOS‐Dem link to clinical decision support tools? What features are needed in a digital app that will host the EMBED‐Care Framework to encourage use and uptake? To understand how person‐centred outcome measures could enhance shared decision‐making.
Workshop 3: Professionals (*n* = 12) and carers (*n* = 7)	Understand how the EMBED‐Care Framework will work with current practice, teams and families.	Online	How can the IPOS‐Dem be completed, including who is best placed to complete the IPOS‐Dem, what happens to the information recorded on IPOS‐Dem and who can access the clinical decision support tools? How do the alerts from the IPOS‐Dem work—who receives them, when should an alert be generated and should alerts be tailored? How could the Framework integrate with current care processes—how does it work with existing patient/carer assessments, how does it work with care planning and how would the app work on paper?
Workshop 4: Carers (*n* = 4)	To gain feedback on the contents of the clinical decision support tools.	Online	What are the strengths and weaknesses of each of the clinical decisions support tools? How can the clinical decision support tools be simplified? How would these work in practice?
Workshop 5: Professionals (*n* = 7)	To gain feedback on the contents of the clinical decision support tools.	Online	What are the positives and negatives of each of the decision support tools? How can the clinical decision support tools be simplified? How would these work in practice?
Workshop 6: People with dementia (*n* = 3) and carers (*n* = 4)	Explore the involvement of people with dementia in the EMBED‐Care framework, including shared decision making. Understand how the EMBED‐Care Framework can be used electronically.	Online	How could the IPOS‐Dem be used on a tablet? What would encourage people to use the Framework? How can the IPOS‐Dem be completed with people with dementia?
Workshop 7: People with dementia (*n* = 3) and carers (*n* = 5)	Explore with people with dementia and carers the EMBED‐Care Framework using an app to understand design and use, including challenges to use.	In person	Training on how to use the digital app and the Framework. Testing usage of the digital app and the Framework using think aloud and cognitive interviewing methods—guided by specific tasks to complete to help understand accessibility.
Workshop 8: Professionals (*n* = 18)	Explore with professionals, people with dementia and carers the EMBED‐Care Framework using an app to understand design and use, including challenges to use.	In person	Testing usage of the digital app and the Framework using think aloud and cognitive interviewing methods—guided by specific tasks to complete to understand accessibility. Role play using vignettes to practice use of the digital app and the Framework.
Workshop 9: Professionals (*n* = 4) and carers (*n* = 3)	To understand how the EMBED‐Care Framework could enhance shared decision‐making for people living with dementia at home.	Online	Exploring how the different components of the Framework could enhance shared decision‐making, including when and how to use it.
Workshop 10: Professionals (*n* = 4) and carers (*n* = 3)	To identify the requirements to use the Framework to support shared decision making for people living with dementia at home.	Online	Exploring the resources and support required to use the Framework for shared decision‐making
Workshop 11: Professionals (*n* = 7)	Explore with care home professionals what might influence uptake of the Framework in care homes.	Online	What might influence use of the Framework in your setting? What barriers might inhibit use?
Workshop 12: Professionals (*n* = 5)	Explore with care home professionals how we might successfully engage individuals in using the Framework.	Online	How can we engage end users? What might successful training and support look like?

#### 2.7.1 Format of Workshops

Our approach to workshops was blended, spanning a continuum of methods as described by Davis and colleagues, including in person (*n* = 2), online (*n* = 6) and supplemented with asynchronous methods [50]. Asynchronous methods included participants providing written feedback through email for those who were not able to join online, felt uncomfortable joining a group/online and to provide additional feedback between workshops. This approach allowed us to be agile and tailor different approaches to fit different people, maximising partnership and collaboration [51]. This was particularly important due to COVID‐19 social distancing at the time but also as it enabled busy health and social care teams to join online with no need to travel to a venue and disrupt their working day further. We used a combination of modified nominal group processes [52] and think aloud methods [53], which supported problem‐solving and group‐based discussions. To ensure accessibility of workshops for people with dementia, the in‐person workshop was held in their usual meeting place where they already met as a group. We shared information in advance and provided a summary at the end of both sessions. We ensured that additional facilitators attended these workshops so that we could provide support either on a one‐to‐one basis or two‐to‐one. We ensured regular breaks and maintained a pace that matched the groups' preferences. These provisions were well received and ensured fruitful sessions. We received feedback that the use of colour and formatting of some materials needed refinement for people with dementia, which affected perception.

#### 2.7.2 Content of Workshops

All workshops followed a similar process: 1) short presentation by a member of the research team on the project's aims, progress and aim of the workshop; 2) small group work in breakout rooms (small group work for in‐person workshops) with focused questions and thoughts recorded using Google JamBoard; 3) feedback and discussion among larger group; and 4) summing up and key take‐home messages for the research team to revise the intervention.

**Figure 1:**
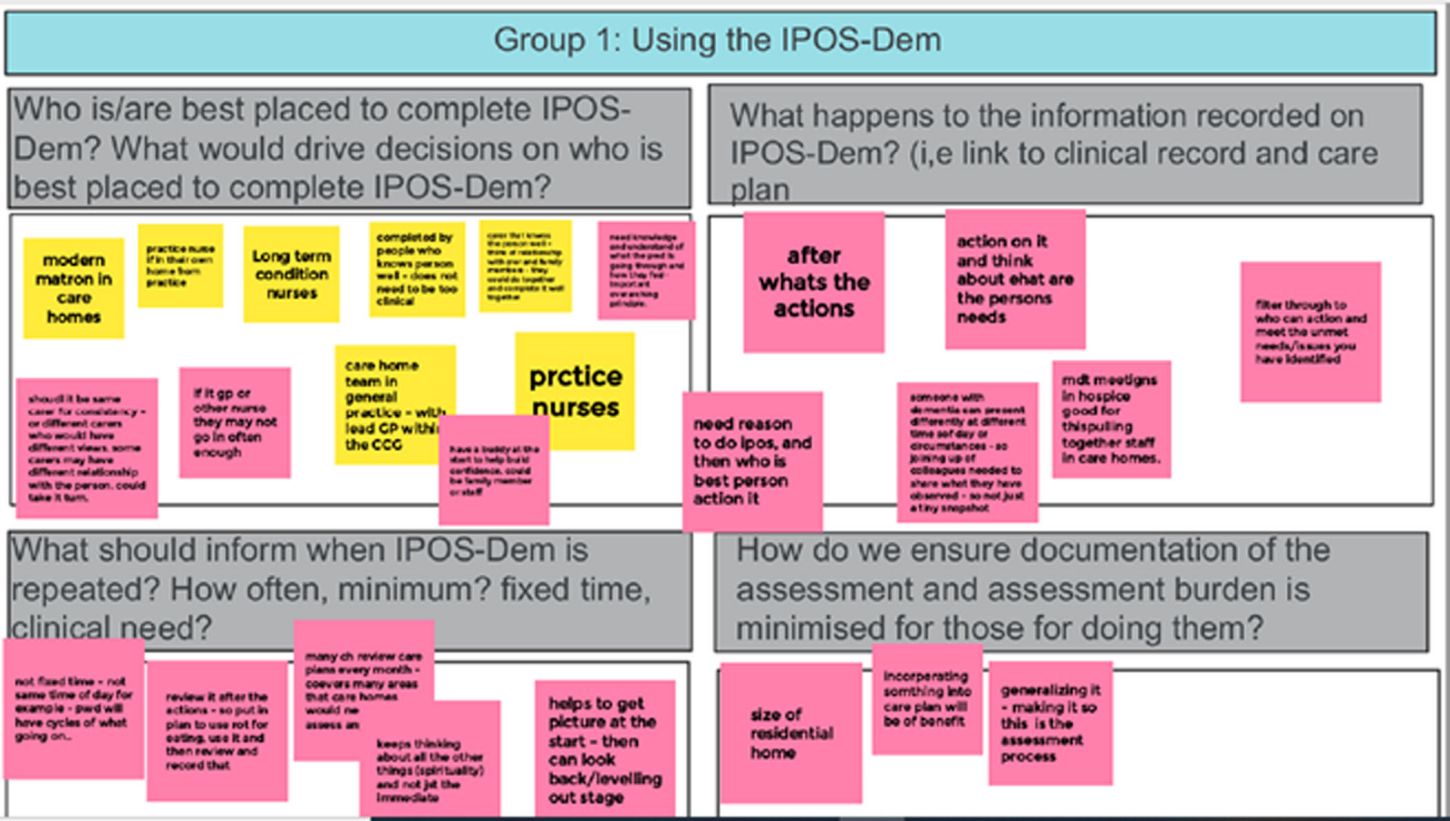
Example Google Jamboard.

The virtual workshops were conducted on Zoom and used a mixture of activities and digital tools to support discussion and engagement. Where possible, materials were sent in advance of the workshop either via email or by post. This enabled people to have a physical connection with the workshops and discussions, whilst also reflecting on their thoughts either before or after the workshops [51]. We ensured that we considered using activities and tools that were interactive and engaging, and placed participants into smaller groups for discussions [54]. For example, we used focussed questions, hand‐outs, sorting and prioritisation activities and scenarios/case studies/storytelling, which can help improve connection among participants, and hence to provoke reflections, ideas and generate discussion [55]. Google Jamboard was used to record participant discussion and create digital post it notes; this also allowed those who did not wish to speak to record their thoughts (see Figure 1). We utilised the breakout rooms and chat function to facilitate discussion and encourage input in smaller groups. Two final workshops that focussed on user testing the Framework prototype were conducted in person.

Across workshops, participants were asked to consider and comment on the evidence base, and reflect on their experiences, to inform the content and design of the intervention. Workshops followed each stage of the design process and considered the relevance and accuracy of the Framework, how the Framework will be used, content and focus, aspects of implementation, including involvement of people with dementia, fit with current practice and use of the digital app. They were encouraged to consider common key factors that may influence assessment and decision‐making in vulnerable or marginalised groups. This encouraged the clinical decision support tools to capture subtleties in decisions, by considering differences among carers and the needs of individuals. We presented the IPOS‐Dem to consider how it can be used as part of the intervention, including frequency of assessments and how it should support and inform decision‐making. As our intervention was developed and refined, we sought feedback on acceptability and resources required (e.g., training and manuals) to deliver and use the Framework.

Participants were asked to consider the implementation of the Framework into the real world, including how technology can be used to support implementation. We worked with Aetonix, a specialist digital health provider, who we met regularly between co‐design workshops to explore how stakeholders can use the application that would deliver the Framework.

In between workshops, the discussions, information and key decisions noted in the sessions were used to iteratively develop and refine an initial prototype intervention by the research team, supported by our Patient and Public Involvement (PPI) members. This initial prototype was used to inform subsequent workshops. The training and support package was designed and reviewed in a final workshop.

### 2.8 PPI

Throughout, we were guided by our PPI group consisting of three people with mild dementia, including younger onset dementia, and seven family carers. PPI helped to develop workshop content and format, provided feedback on the emerging Framework and guided interpretation of findings. Specifically for intervention development, we hosted two PPI workshops and comments were also provided by email on the Framework. We conducted a final user testing workshop with the PPI members in September 2022.

### 2.9 Ethics Approval and Consent to Participate

This study received ethical approval by the London Queen Square Ethics Committee and Health Research Authority (HRA) on 01.06.2022 (Ref20/LO0295). Written informed consent was obtained from all participants.

## 3 Findings

### 3.1 Participant Characteristics

Participant characteristics are described in Table 5 below, and numbers in each workshop are shown in Table 4.

**Table 5:** Participant characteristics.

	Family carers (*n* = 18)	Practitioners (*n* = 55)
Age: Median (range)	63.5 (42–81)	47 (28–66)
Gender		
Women (*n*)	16	49
Marital status (*n*)		
Single, never married/in civil partnership	4	8
Cohabiting, married or in a civil partnership	12	42
Other	2	2
*Missing*	0	3
Ethnicity		
White (English or other)^a^	18	45
Black (African, Caribbean or other)	0	3
Asian (Pakistani, Sri Lankan or other)	0	2
Other	0	2
*Not reported or missing*	0	3
Level of education		
Degree or equivalent	10	43
General certificate of secondary school or general certificate of education	5	2
Other qualifications	3	7
*Missing*	0	3
Practitioner role		
Nurses^b^		28
Care home managers and leads		4
General practitioners		2
Digital lead		
Other^c^		15
*Missing*		3

^a^ ‘White other’ included Irish, Welsh, Scottish, Northern Irish, German, Irish, South African and Portuguese.

^b^ Nurses included clinical nurse specialist (including leads and consultants), community nurse specialists, dementia nurse specialists, community nurses (including leads and consultants, nurse liaisons, palliative care nurse, registered nurses and senior staff nurses, dementia outreach nurses).

^c^ Other included nursing assistants, dieticians, patient and public involvement coordinator, field support supervisors, Domiciliary care managers and staff members, clinical psychologists, advanced clinical practitioners, dementia support officers, digital technology specialists, palliative medicine consultants and hospice education lead.

### 3.2 Overview of the Intervention

The EMBED‐Care Framework has been developed for use by professionals in care homes (residential) and in the community, as well as for the person with dementia and family carers. The Framework consists of five main components:

1. IPOS‐Dem to enable comprehensive routine assessment of symptoms and concerns.
2. The alert system linked to the IPOS‐Dem automatically generates an alert based on the score. IPOS‐Dem items that score 3–4 will result in a red alert and those that score 1–2 will result in an amber alert. Items scoring 0 will show as green.
3. Priority setting using the alert system and scores from the IPOS‐Dem to prioritise care of symptoms and concerns.
4. Clinical decision support tools linked to the IPOS‐Dem to support management and decision making about symptoms and concerns raised from the IPOS‐Dem, and priority setting.
5. Training package consisting of in‐person or remote training workshop, manuals, animated bite‐size videos and local champions facilitating the intervention.

**Figure 2:**
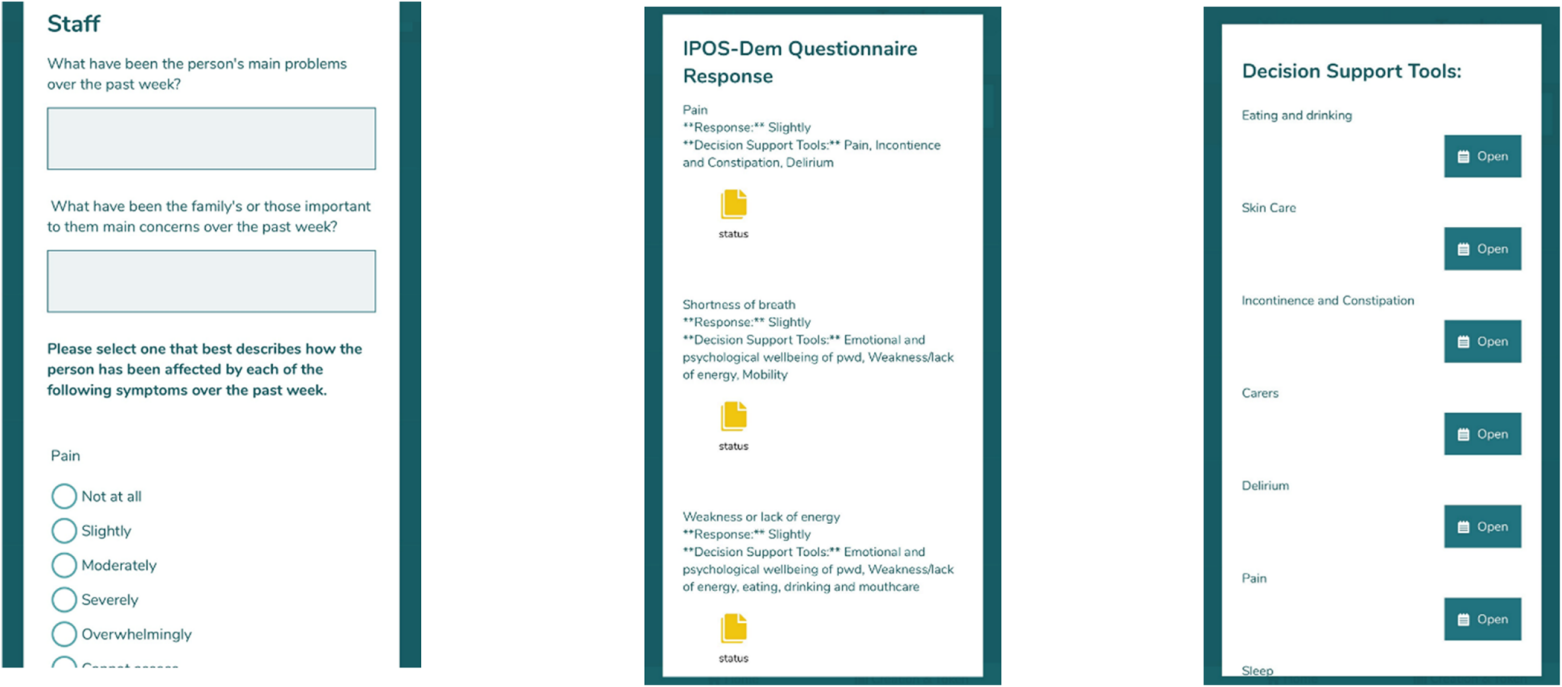
aTouchAway delivery of the Framework.

**Figure 3:**
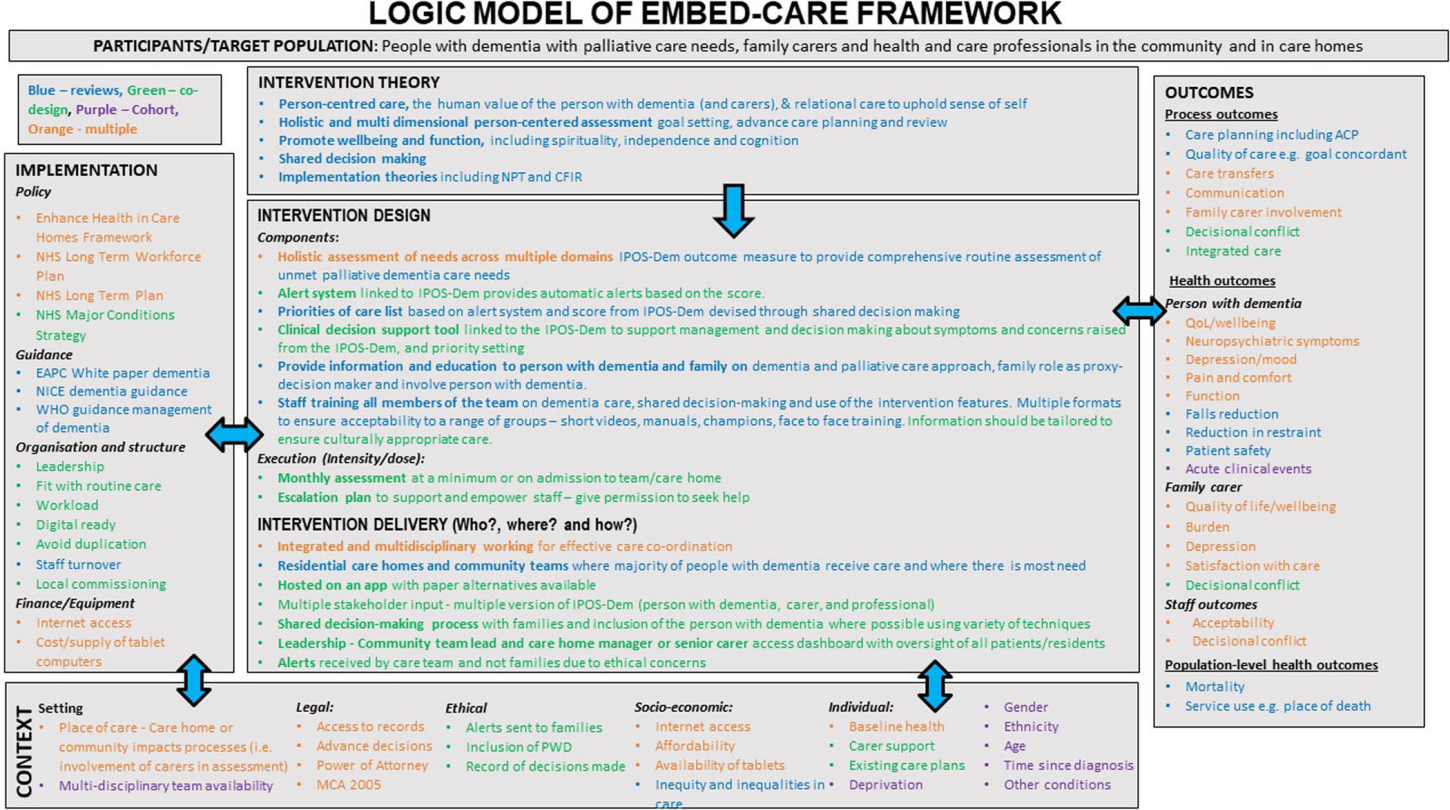
EMBED‐Care Framework logic model [37].

**Figure 4:**
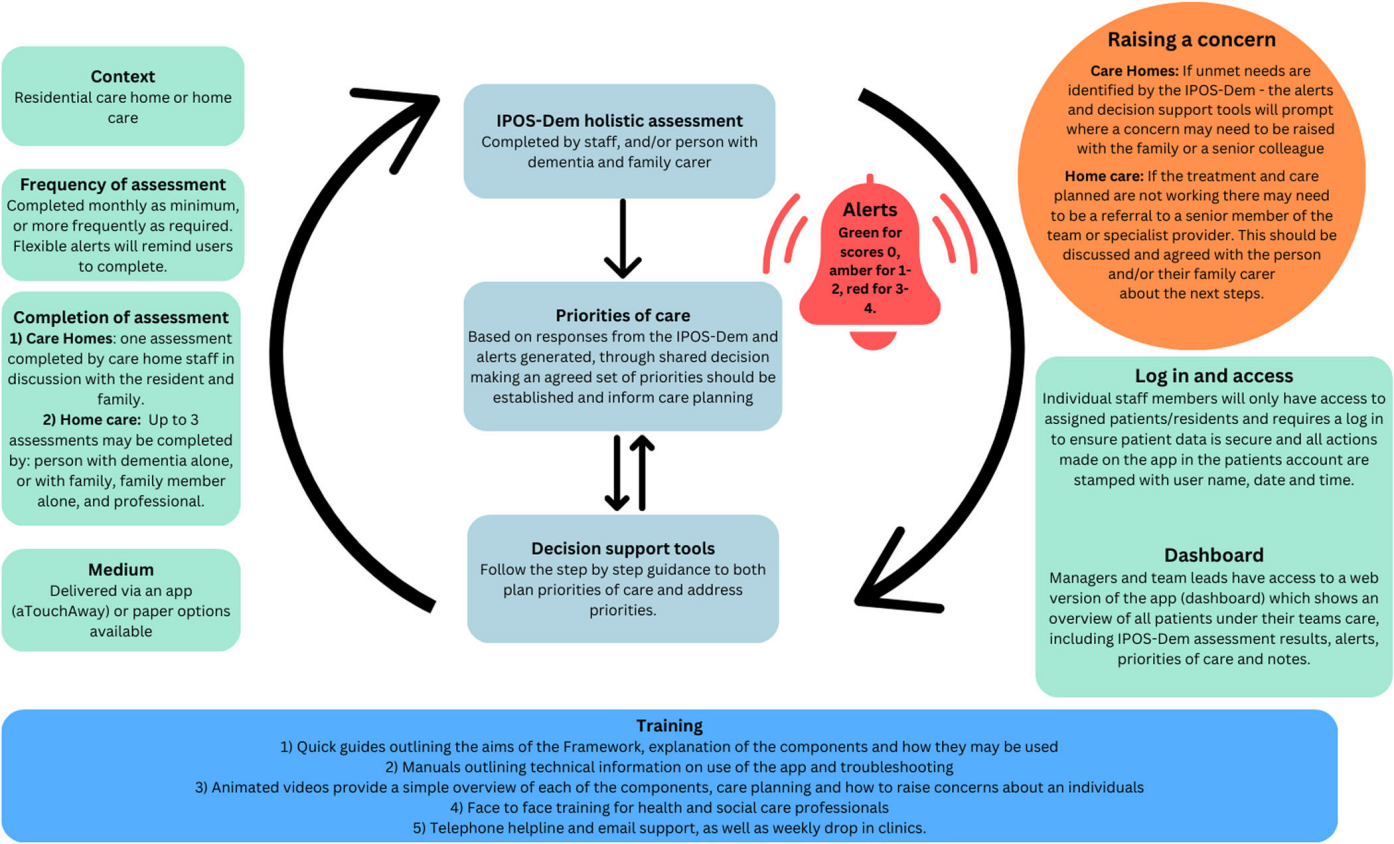
Intervention overview and flow.

The EMBED‐Care Framework is delivered through an app delivered by aTouchAway, with elements available in paper version if preferred. The app can be viewed on a smart phone or tablet (see Figure 2). A web‐based version (dashboard) is available for professionals to view all their patients/residents, including results of the IPOS‐Dem assessment, allowing them to track symptoms over time, identify those who have symptoms and concerns that need addressing. The dashboard can be used by a senior member of staff who is coordinating the team and care—for example, in a care home, the senior nurse in charge or care home manager. Our logic model refined from the co‐design workshops and flow of the Framework are shown in Figures 3 and 4, respectively.

### 3.3 Focus of the Intervention

We presented from our earlier work the key palliative care needs and symptoms in dementia to the participants and asked them to consider what should be the focus and priorities within the Framework. However, all needs/symptoms were felt to be important to deliver a holistic approach to care. Importantly, participants highlighted that this should be a practical tool that enhances and supports care. Participants identified four key priorities for the Framework to focus on 1) Holistic approach—looking beyond the individual symptoms and concerns to ensure that the individual as a whole and all their varying needs across physical, psychological, social and spiritual domains were considered. 2) Integrated care through clear communication—supporting communication, within and between teams, with the person with dementia and with family carers. 3) Maximising quality of life (QOL)—many of the symptoms that would be addressed as part of the intervention should have the aim of maximising QOL, providing the right care at the right time. 4) Managing uncertainty—take a more proactive approach, preparing teams and families to know what to ‘look out for’ and empowering them through information and practical resources.

### 3.4 Holistic Assessment

**Figure 5:**
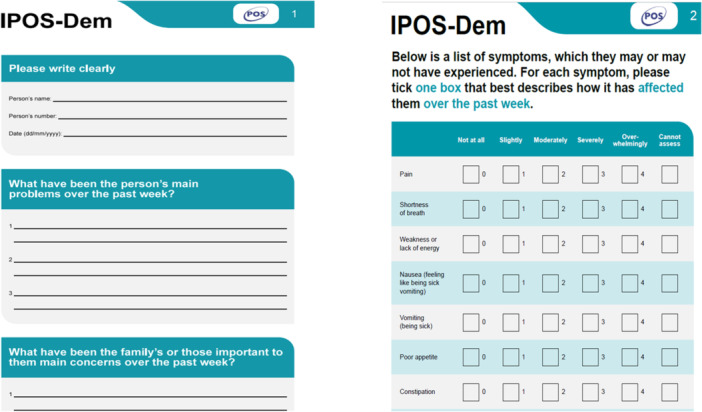
IPOS‐Dem holistic assessment.

The first component of the intervention is the use of the IPOS‐Dem to conduct a holistic assessment of needs and concerns [14] (see Figure 5). Workshop discussions focussed on three main questions about the use of the IPOS‐Dem: 1) who should complete the IPOS‐Dem? 2) When and how often should the IPOS‐Dem be completed? 3) What happens to the results of the IPOS‐Dem? Participants were keen to highlight that the person with dementia should either lead or be included in the discussion/assessment regardless of their capacity or communication abilities. Although there was a desire to have a key person who led assessment and this person remained consistent, such as the GP, participants also felt that this was not feasible. It was more important that the person completing the assessment knew the individual well. This did not need to be the same person each time. Timing was agreed to be important; this included at admission, and then a minimum of once a month, with flexibility to repeat assessments within the month if needs changed. The most important part was to ensure that this was embedded in practice, not duplicating existing work but complementing, aiding and enhancing existing work within the care teams and leading to actions.

### 3.5 Alert System

Participants emphasised that there is a requirement from this Framework for action or change in response to identified symptoms and concerns. An inbuilt alert system is key to linking the outcomes of the IPOS‐Dem to action, providing the right care at the right time. An amber notification is automatically generated if a concern on the IPOS‐Dem scores 1 or 2 (slightly or moderately affected), and red for scores 3 or 4 (severely or overwhelmingly affected). A notification is received on the app and visible on the dashboard. For care home staff, our training encourages them to raise a concern to their senior colleagues or family carers, empowering them to act. Tailoring alerts to the individual was seen as adopting a person‐centred approach and responsive to the person. Alerts can be tailored for everyone for some items; for example, the mobility item on the IPOS‐Dem should consider the normal scoring range for that person. Importantly, the IPOS‐dem asks how much a person is affected by a symptom, so the mere presence of a symptom will not prompt an alert if it is not affecting the individual. This was in response to concerns from professionals that no modification would lead to constant alerts for someone who is bed bound, for example, and would lead to all alerts for that individual potentially being silenced and ignored. There was disagreement about tailoring of other alerts, with some arguing the need to be able to tailor the intervention, and others querying how to determine a cutoff score that may lead to some alerts being missed that are important or indicate a small but important clinical change. Some carers and our PPI group were keen for carers to also receive alerts via the app, especially if being used in a care home setting. However, it was felt by the research team and professionals that this was unethical as the carer may receive an alert that they can do nothing about, except worry. In the feasibility study, these considerations will be explored in depth.

### 3.6 Clinical Decision Support

To respond to the needs identified in the holistic assessment, clinical decision support tools were co‐designed. Discussions in workshops as to who was responsible for acting on the unmet needs often linked back to the content of the clinical decision support tools to guide this decision as different needs could be met by different people. However, ultimate responsibility for overarching processes needed to be established. Unfortunately, due to the fragmented health and care systems and variations in commissioning across England, this was not possible to standardise. In care homes, there was a consensus that this should be led by a nurse in charge, or a clinical team linked to the care home, such as community matrons. For those in the community, it was likely to be the community nursing team or GP. The key was flexibility in the delivery of this Framework and discussions with and within individual teams using the Framework as to how it can be adapted to the local context and needs of the teams/services.

**Figure 6:**
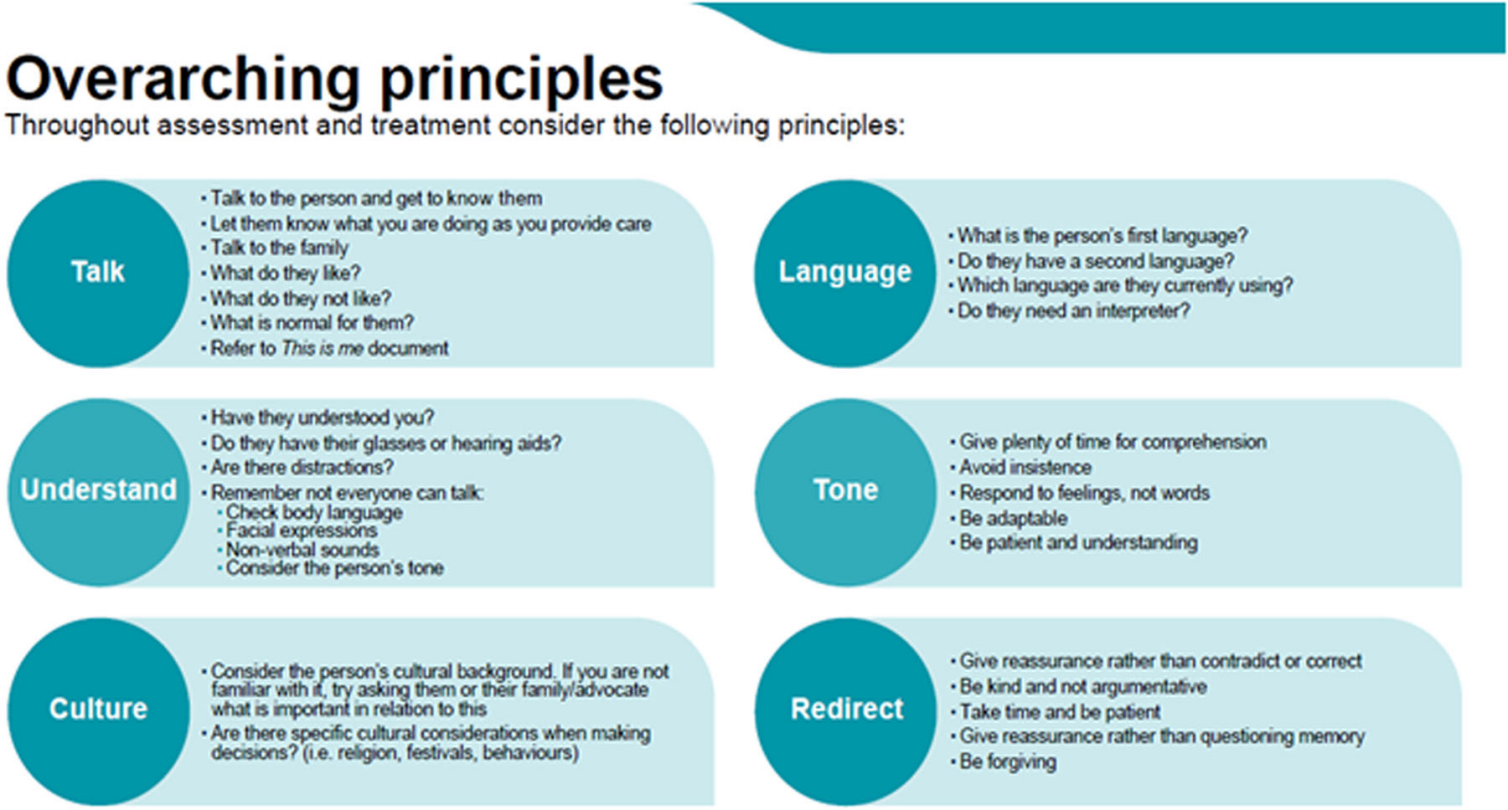
Example of clinical decision support tools—overarching principles.

Twelve clinical decision support tools were created aligned to each item of the IPOS‐Dem and the key palliative care needs identified by the evidence and confirmed by participants in the first workshop. The clinical decision support tools start with an overarching principles summary that considers key points to consider throughout assessment and treatment, including communication and culture (see Figure 6), followed by assessing the care environment.

After this introduction, there are 11 needs‐ or symptom‐specific clinical decision support tools: 1) spiritual, identity and well‐being; 2) delirium; 3) eating, drinking and mouthcare; 4) pain; 5) skin care; 6) sleep; 7) constipation and incontinence; 8) weakness and lack of energy; 9) mobility; 10) maintaining emotional and psychological well‐being; and 11) carer well‐being.

**Figure 7:**
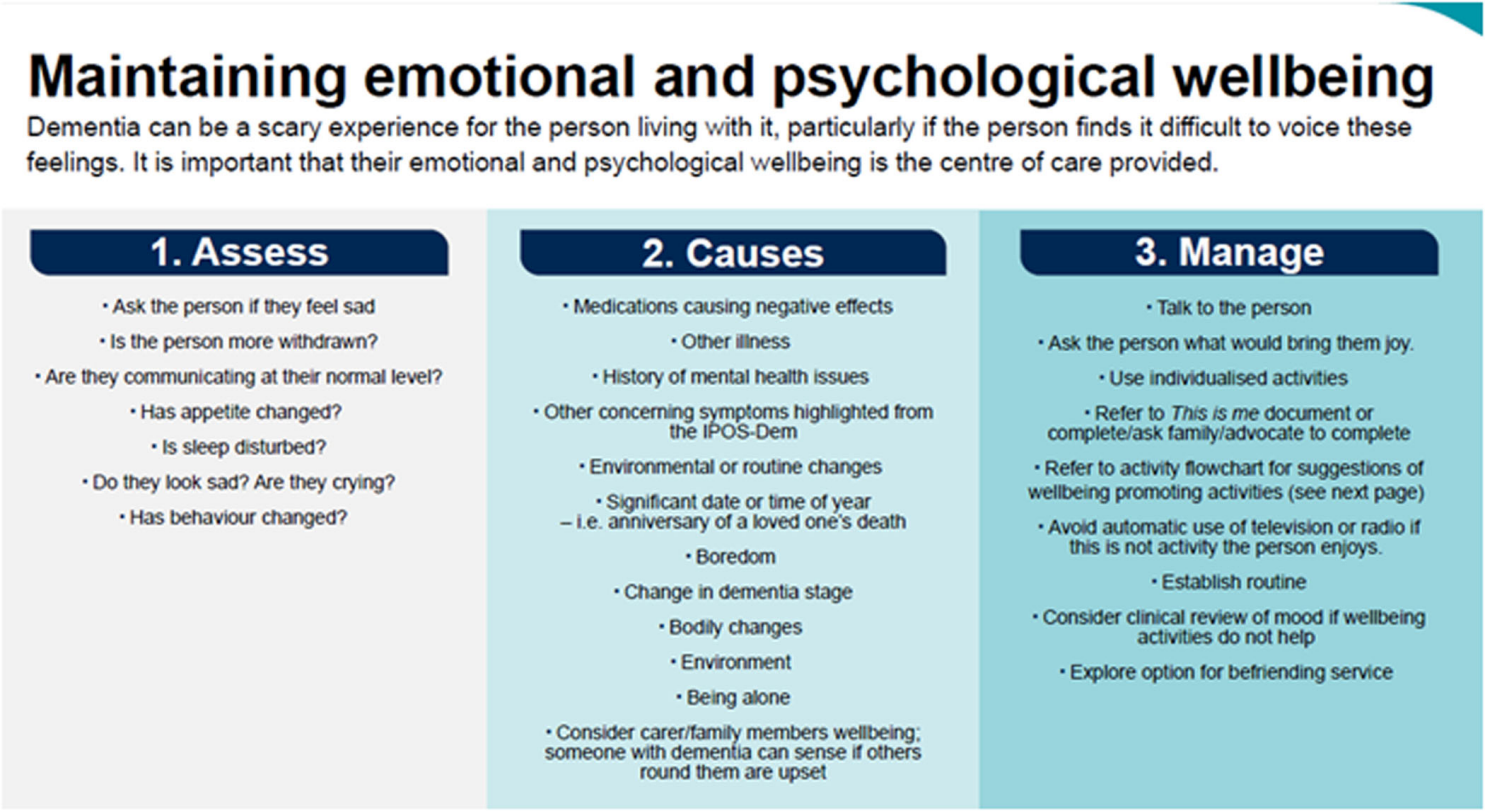
Example of format of clinical decision support tools.

As can be seen in Figure 7, the clinical decision support tools are presented simply and clearly with short prompts to stimulate thinking. The majority follow a box format with three phases of: assess, causes and manage. This reflects previously published decision support tools that follow the principles of rules of thumb that make implicit tacit knowledge of best practice and evidence, which has been collated over many years of experience, explicit in simple form for even novice users to be supported and act [22, 23].

### 3.7 Priority and Goal Setting

The discussion of the use of the IPOS‐Dem highlighted that there may be many unmet needs identified and varying responses (e.g., from professionals, carers and individuals completing the IPOS‐Dem separately) to different needs that would need to be managed. Users of the Framework will be able to create a priority of care list or a summary of discussions to inform the care plan guided by the clinical decision support tools. We provide tips in the training components, including the manual on how to support joint working and managing different answers or priorities. Shared decision‐making was a key component of this intervention and study; it was explored in depth and reported separately due to the complexity and importance of the topic [56].

### 3.8 Training

To support the use and delivery of the Framework, a comprehensive and tailored training package was co‐designed and created. Manuals for the Framework were constructed, including an explanation of the Framework and its components, who the Framework is aimed at, using the different components, managing conflicting priorities among stakeholders and finally additional information on principles of palliative care and dementia. To complement the manuals, short video animations were created to explain and demonstrate the main components. Through the app, additional training resources are available, including glossary and links to third‐party resources. Within each of the sites for testing the Framework, in‐person training is available and dedicated champions support use and troubleshooting.

## 4 Discussion

We co‐designed a novel digital intervention that links components across holistic assessment, person‐centred outcome measures and shared decision‐making with decision support tools. Previous studies in these fields have worked in isolation, with much of the holistic assessment and person‐centred outcome measures lacking solutions to manage the identified unmet needs from assessment with patients [14]. Similarly, pragmatic and applied clinical decision support tools, including rules of thumb developed for use in palliative dementia care, lack a comprehensive and holistic assessment to first identify unmet needs [19, 22]. Our intervention is the first in this field to link together holistic assessment and clinical decision support tools. Underpinned by theory from implementation science [57, 58], shared decision making [16] and person‐centred care [59], we synthesised evidence from a range of sources to co‐design our framework with people with dementia, family carers and health and care experts.

While supporting holistic assessment and decision‐making, the Framework also addresses some of the key challenges with the provision of palliative dementia care, including communication and integrated care [60, 61]. UK health and care systems are fragmented, and this is particularly challenging for those with dementia, who often require the support of multiple services simultaneously, leading to more transitions, longer stays in hospital and poor care [62, 63]. The Department of Health and Social Care, together with NHS England and the government, are prioritising the development and implementation of new ways to deliver care, including digital supported approaches, so that more people get the best possible care. The NHS 5 Year Forward View [64], NHS Long Term Plan [65], NHS Long Term Workforce Plan [66], Health and Social Care White Paper [67], Major Conditions Strategy [68] and Framework for Enhanced Health in Care Homes [69] call for a holistic approach to care that is joined up between different teams and services. The EMBED‐Care Framework offers an approach to join the gaps between services and teams and, in doing so, improving communication. The digital element makes it possible for multiple teams and individuals to view the results of the holistic assessment and discuss and agree priorities, which are then acted upon in a shared decision‐making approach.

Previous work has concluded how clinical decision support tools break down complexity of dementia care [22]. We argue that this Framework breaks down more than just the decision‐making process but covers the entire journey from assessment through to decision, action and review. For many professionals, the complexities associated with dementia care and the unmet needs that an assessment may reveal can feel overwhelming. Patients may have many unmet needs identified by an assessment. This can create feelings of being overwhelmed and cause further uncertainty, leading to inaction and potentially patient harm [70]. Prioritisation can help manage this overwhelming feeling and empower staff and family carers, to ensure that needs are considered and action is taken, thus bridging the implementation gap between assessment and action. This is an important component that is often neglected in interventions and clinical practice.

In developing this Framework, we worked closely with a digital provider and our stakeholders to deliver the Framework as a digital intervention. Delivering the Framework on a digital application future‐proofs the Framework among a growing digital landscape in health and care delivery. The digital platform enables information, including the holistic assessment and priorities of care, to be shared among staff and different teams. During and since the COVID‐19 pandemic, there has been a surge in the use of digital technology in care; in particular within palliative care teams, technology has been used to increase communication among teams and with families [71]. Recent work has demonstrated that the use of digital technology in care homes to share information about residents resulted in fewer hospital attendances, admissions and reduced length of stay, as well as cost savings for the NHS [72].

### 4.1 Implications for Clinical Practice, Policy and Research

The Framework has potential to provide a more integrated approach to palliative dementia care, if adopted by various teams. The EMBED‐Care Framework is ready for testing in a feasibility study with residential care homes and community nursing teams in England. Careful consideration is needed to ensure if feasible and acceptable, the most efficient way to test the Framework while minimising delay to implementation, given the great need for support in this field. Interoperability of the Framework is a key issue across the various electronic systems in various services and localities.

The Framework has the value of consisting of a person‐centred outcome measure for holistic assessment that accounts for symptom combinations, which is linked to decision support tools. Using a person‐centred outcome measure allows for proactive assessment and monitoring to identify when a person may be deteriorating, which goes beyond the scope of many holistic assessments. There is evidence from palliative care that the use of person‐centred outcome measures facilitates decision‐making and supports communication, symptom identification and ongoing monitoring [73].

In co‐designing our Framework, we used a variety of methods to ensure transparency and rigour in our approach. The matrix approach to synthesise evidence has been used previously [20, 31], in addition to offering transparency, it provided a structure to manage large amounts of evidence and findings, and we would recommend this for future intervention development. However, there is a need to balance how much evidence is used, as this can lead to being overwhelmed even when using a structured synthesis approach as we did.

The pandemic encouraged us to look at alternative methods to our traditional approach to in‐person co‐design workshops, including the use of digital tools such as Google JamBoard for online meetings and workshops. We also embraced various asynchronous and synchronous approaches, including email/written feedback and individual meetings, which was an effective way of including a wide variety of stakeholders and should continue in the post‐pandemic era.

### 4.2 Strengths and Limitations

People with dementia are often excluded from palliative and end‐of‐life care research, and during the pandemic, online meetings were particularly challenging for people with cognitive impairment. However, our study reflects true co‐design, with inclusion of people with dementia from both our PPI group and DEEP workshops. Additional sessions with people with dementia would have strengthened this further, as the workshops were conducted in the mid to late stages of development. Despite this, we gained feedback from people with dementia from our PPI group throughout the planning and development stages. We aimed to recruit a diverse pool of participants with regard to age, gender and ethnicity/language/culture/religion. However, this was challenging despite a variety of different recruitment routes and attempts. As the initial co‐design workshops were online (due to COVID‐19) and the latter were in person in London, we may have excluded certain groups, including those who are not able to use the internet, who tend to be people from already marginalised groups. Many of our recruitment methods also focussed on those who were already seeking help. We know that those who are less likely to seek help are those from minority backgrounds, and non‐White British cultures, therefore our recruitment methods may have missed those who are most under represented. Future work should look to work closely with community partners to maximise diversity. We included stakeholders from a variety of disciplines, including general practice, geriatrics, palliative care, psychiatry, home care, care homes and community nursing, to consider use across different teams and maximise potential for broad uptake. Although we included our digital technology company in discussions throughout the development of the EMBED‐Care Framework, they were not included in co‐design workshops. Their inclusion from the start as part of the workshops may have helped manage expectations about the possibilities of the digital technology and helped focus discussions.

## 5 Conclusions

We have developed a novel digital palliative dementia care Framework that links holistic assessment and clinical decision support, with important components to bridge the gap between these two ends of the care spectrum, and training package for use in community and social care settings. Methodologically, we provide a clear overview of an approach to co‐design to inform subsequent co‐design intervention development, alongside presenting the core components of the EMBED‐Care Framework.

## Author Contributions

**Nathan Davies:** methodology, conceptualisation, funding acquisition, writing–original draft, writing–review and editing, formal analysis, data curation. **Elizabeth L. Sampson:** conceptualisation, funding acquisition, writing–review and editing. **Jesutofunmi Aworinde:** writing–review and editing, data curation, project administration. **Juliet Gillam:** data curation, project administration, writing–review and editing. **Charlotte Kenten:** data curation, project administration, writing–review and editing. **Kirsten Moore:** writing–review and editing, funding acquisition, conceptualisation. **Bethan Phillips:** data curation, writing–review and editing. **Catherine Harvey:** project administration, data curation, writing–review and editing. **Janet Anderson:** writing–review and editing, funding acquisition, conceptualisation. **Jane Ward:** funding acquisition, writing–review and editing, formal analysis. **Catherine J. Evans:** data curation, writing–review and editing, funding acquisition, conceptualisation, formal analysis. **Clare Ellis‐Smith:** data curation, formal analysis, writing–review and editing, funding acquisition, conceptualisation.

## Ethics Statement

This study received ethical approval from the Queen Square Research Ethics Committee and Health Research Authority (HRA) on 01.06.22 (REC Ref20/LO/0295). Written informed consent was obtained from all participants.

## Consent

All participants provided consent to publish.

## Conflicts of Interest

The authors declare no conflicts of interest.

## Data Availability

The data sets used and/or analysed during the current study are available from the corresponding author on reasonable request.
